# Hepatic Encephalopathy: From the Pathogenesis to the New Treatments

**DOI:** 10.1155/2014/236268

**Published:** 2014-06-04

**Authors:** Juan Cordoba

**Affiliations:** ^1^Departamento de Hepatología, Hospital Vall d'Hebron, Passeig Vall d'Hebron 119-129, 08035 Barcelona, Spain; ^2^Departamento de Medicina Interna, Universidad Autónoma de Barcelona, Spain; ^3^CIBERehd, Instituto de Salud Carlos III, Madrid, Spain

## Abstract

Hepatic encephalopathy is a frequent and serious complication of liver cirrhosis; the pathophysiology of this complication is not fully understood although great efforts have been made during the last years. There are few prospective studies on the epidemiology of this complication; however, it is known that it confers with high short-term mortality. Hepatic encephalopathy has been classified into different groups depending on the degree of hepatic dysfunction, the presence of portal-systemic shunts, and the number of episodes. Due to the large clinical spectra of overt EH and the complexity of cirrhotic patients, it is very difficult to perform quality clinical trials for assessing the efficacy of the treatments proposed. The physiopathology, clinical manifestation, and the treatment of HE is a challenge because of the multiple factors that converge and coexist in an episode of overt HE.

## 1. Introduction

Hepatic encephalopathy (HE) is a disturbance in the central nervous system (CNS) function due to hepatic insufficiency or portal-systemic shunting. HE causes a spectrum of neurologic manifestations that develop in association with different liver diseases [1]. A common link is the potential reversibility of the neurologic manifestations once the abnormality of liver function is corrected. The shunting of blood from the portal venous bed into the systemic circulation is considered a key element of HE. There are a series of neurological disorders associated with liver disease that are not considered HE. This disorders may share a common pathogenetic mechanism, for example, brain and liver damage caused by alcohol or copper (Wilson's disease). HE must also be differentiated from neurologic disturbances caused directly by bilirubin accumulation (kernicterus), cerebral hemorrhage secondary to disorders of coagulation caused by the liver disease, or other abnormalities that are not secondary to liver failure.

The nomenclature that several authors have used for HE is confusing. For this reason several efforts have been made to reach a consensus, especially for the design of clinical trials [2]. Despite this limitation, from a clinical perspective, HE is generally classified according to the underlying liver disease and the evolution of the neurological manifestations (Table 1). The most frequent liver disease is cirrhosis, usually accompanied by extrahepatic portal-systemic shunts (spontaneous or surgical). HE also can be seen in acute liver failure, where it constitutes the hallmark of the disease. In rare cases, HE develops in the absence of any sign of parenchyma liver disease and is caused solely by portal-systemic shunting of congenital or surgical origin.

The neurologic manifestations are variable. The most distinctive presentation is an acute episode characterized by the sudden onset of confusion that can evolve into coma. Neuromuscular abnormalities are common, with the most characteristic one being the presence of asterixis; pyramidal signs may also be present. The term chronic hepatic encephalopathy is reserved for patients who have frequent episodes of episodic encephalopathy or persistent cognitive (memory loss, confusion, and disorientation) or neuromuscular disturbances (tremor, apraxia, and rarely paraplegia). Minimal hepatic encephalopathy corresponds to those neurologic manifestations that are not obvious on clinical examination but are detected by the demonstration of abnormal neuropsychological or neurophysiological tests [3].

## 2. Pathogenesis

### 2.1. General Aspects

Different hypotheses have been proposed to explain the changes in mental state that occur in HE. Ideally, such a theory should explain the relation between liver and neurological abnormalities. However, establishing such relations is difficult, in part, because of limitations in the methods available to study brain function in humans and also due to an insufficient knowledge of the neurobiological basis of behavior. Nevertheless, there is a generalized consensus that the validation of a hypothesis should explain the mechanism of action of a precipitating factor and how specific therapies improve HE [4].

A common pathogenetic notion is that HE is caused by substances that under normal circumstances are efficiently metabolized by the liver, rather than an insufficient production of substrates that could be essential for neurologic function. Under this light, portal-systemic shunting plays a critical role, as the main impact of this circulatory disturbance is on the concentration of gut-derived substances that are highly cleared by the liver. Studies of cross-perfusion in animals with experimental HE and liver support systems in humans have shown that clearance of toxic substances present in the blood is more important to improve mental function than the synthetic capacity of the support system [5]. In patients with liver disease, these toxic substances reach the systemic circulation as a result of portal-systemic shunting or reduced hepatic clearance and produce deleterious effects on brain function. Once the toxic substances are in neural tissues, a large number of neurochemical changes occur that affect multiple neurochemical pathways, each affected to a variable extent [6].

Historical hypothesis have ranged from* unifying theories* [7] to the notion of HE as a* multifactorial process* [8]. As in other metabolic encephalopathies, general neuronal dysfunction results in abnormalities of consciousness. However, in contrast to other conditions that affect consciousness (such as hypoglycemia), where neuronal function is primarily affected, the cell that is abnormal in HE corresponds to the astrocyte that shows characteristic changes in morphology and function. This feature has led to the interpretation that HE is mostly a disturbance of consciousness secondary to altered communication between the astrocyte and the neuron [9]. Alternative views, such as the GABA (*γ*-aminobutyric acid) theory, explain the spectrum of HE through the direct effect of one toxin on a key aspect of neurological function [10]. Other paradigms arise from the experimental observation that different toxins enhance the negative effects of ammonia on consciousness (*synergistic theory*) [11]. This concept has been expanded to include the contribution of systemic inflammation to hepatic encephalopathy [12], as well as alterations in the cerebral blood flux and in the oxidative metabolism [13].

### 2.2. Ammonia

Ammonia is considered an important factor in the pathogenesis of HE. The data that support the involvement of ammonia are as follows. (a) Ammonia is produced by the gut, and an important amount is of bacterial origin [12]. (b) The concentration of ammonia in portal blood is high, and a high degree of extraction occurs in the liver [14]. (c) Concentrations of ammonia are high in the systemic circulation and in the cerebrospinal fluid of patients with HE [15]. (d) Precipitating factors cause elevations in the blood level of ammonia or result in exposure of brain tissue to ammonia [12]. (e) Strategies that decrease the level of blood ammonia are of benefit [16].

Ammonia is generated in different tissues from the breakdown of amino acids and other nitrogenous substances. Under normal physiological conditions, ammonia enters the portal circulation from the gastrointestinal tract, where it derives from colonic bacteria and from the deamidation of glutamine in the small bowel. Traditionally, absorption was viewed as the result of passive diffusion; more recent studies indicate the presence of specific ammonia transporters [16]. Regardless of the mechanism of absorption, ammonia reaches high concentration in the portal blood and undergoes a high first-pass hepatic extraction (−80%). In the liver, ammonia is transformed in the periportal hepatocytes into urea (a high-capacity and low-affinity system) and in the centrovenular hepatocytes into glutamine (a low-capacity and high-affinity system). Urea is quantitatively the most important product of ammonia metabolism and elimination. Circulating urea diffuses into the intestine (−40%), where it undergoes hydrolysis into ammonia through ureases present in colonic bacteria. Urinary elimination of nitrogen in the form of urea is a route of ammonia disposal from the organism.

In addition to the intestine and the liver, kidney and muscle contribute to regulating the arterial ammonia level [17]. In muscle, ammonia is transformed into glutamine through the action of glutamine synthetase. Experiments in normal volunteers showed that 50% of injected ^15^N-ammonia is removed by the muscles [18]. The ability of the muscle to “fix” appreciable amounts of blood-borne ammonia becomes important to regulate arterial ammonia in case of liver failure and highlights the importance of maintaining an adequate muscular mass in patients with HE. It is generally accepted that at rest skeletal muscle is an ammonia-consuming organ. However, during moderate to heavy exercise, the muscle releases ammonia [19]. The kidneys generate ammonia from the deamination of glutamine, a step involved in the regulation of arterial and urinary pH. A small fraction of renal ammonia is released into the systemic circulation; urinary ammonia excretion may be affected by dehydration and increases in conditions of hyperammonemia [20]. Notwithstanding the role of peripheral organs, the main factors resulting in the increase in blood ammonia in liver failure are a decrease in the capability of the liver to generate urea and glutamine and the bypass of first-pass hepatic metabolism via portal-systemic shunts.

**Figure 1:**
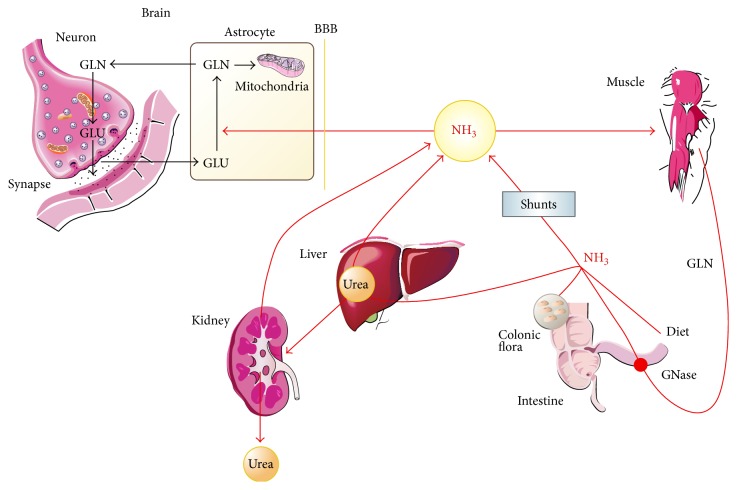
Physiopathology of hepatic encephalopathy. Cordoba Semin Liv Dis 2008. GLN: glutamine; GLU: glutamate; GNase: glutamine synthetase; BBB: blood brain barrier.

Patients with HE have an increased diffusion of ammonia into the brain [12], though recent studies using sophisticated PET techniques have questioned this tenet [13, 21]. Variations in the passage of ammonia across the blood brain barrier may explain the poor relationship between the level of arterial ammonia and the degree of HE; nonetheless, the relation between plasma ammonia levels and cerebral dysfunction seems to be clear in patients with acute liver failure in which plasma ammonia have a direct correlation with the presence of intracranial hypertension and death [22, 23] (Figure 1). This relation may be related to the induction of brain edema by ammonia or simply be an indicator of the severity of liver failure. During HE episodes in cirrhosis, ammonia shows a huge interpersonal variability. This may highlight the importance of additional factors that participate in the pathogenesis of HE. Among them, those more important appear to be inflammatory mediators.

### 2.3. Glutamine

Most recently, glutamine has been shown to have a key role in the brain toxicity induced by ammonia. It has long been accepted that the conversion of glutamate to glutamine, catalyzed by glutamine synthetase, a cytoplasmic enzyme largely localized in astrocytes in brain, represents the principal way of cerebral ammonia detoxification. Much of the newly synthesized glutamine is subsequently metabolized in mitochondria by phosphate-activated glutaminase, yielding glutamate and ammonia. In this manner, glutamine is transported in excess from the cytoplasm to mitochondria serving as a carrier of ammonia. It has been proposed that the glutamine-derived ammonia interferes with mitochondrial function giving rise to excessive production of free radicals and induction of the MPT, two phenomena known to bring about astrocyte dysfunction, including cell swelling [24]. This hypothesis is supported by neuroimaging studies [25] that had shown increased levels of brain glutamine during the HE episode that decreased when the episode had been recovered.

### 2.4. GABA Agonists

Several lines of evidence support the presence of activated GABAergic tone in HE [26]. One of the postulated mechanisms for this effect is the increased availability of agonist ligands of the GABA receptor complex, a key inhibitory neurotransmitter in the brain. The term natural benzodiazepines has been coined for a group of substances of nonpharmacological origin that bind to the benzodiazepine site of the GABA receptor, where they can act as agonist or antagonist. These substances, which are poorly characterized from a chemical and functional perspective, have been reported to be present in a variety of human tissues in normal conditions and to purportedly accumulate in the brain of patients with HE [27]. It has been proposed that natural benzodiazepines with agonist effects on the GABA receptor induce a decrease of consciousness in HE. However, not all the benzodiazepine ligands found in HE have agonist effects on the GABA receptor (e.g., diazepam binding inhibitor). Furthermore, alternative routes of activation of GABA neurotransmission may be present. These include direct and indirect effects of ammonia on the affinity of GABA receptors to its natural ligand [7]. Ammonia may also result in an increased density of peripheral-type benzodiazepine receptors (PTBRs), present in astrocytic mitochondria and whose activation results in the synthesis of neurosteroids, powerful ligands of neuronal GABA receptors [28, 29].

Several arguments have been proposed in favor of a role for natural benzodiazepines in HE. The most relevant is the observation of an improvement of mental state after the administration of flumazenil (a benzodiazepine receptor antagonist) in some patients with advanced stages of HE that have not consumed benzodiazepines of pharmacological source [30]. However, the beneficial effects of flumazenil, usually mild and transient, are only seen in a subgroup of patients. One of the main limitations of this theory is the lack of an explanation of the mechanism by which natural benzodiazepines increase in HE. A study in rats with experimental HE showed generation in the intestinal flora of precursors of natural benzodiazepines [31]. These precursors would be transformed into natural benzodiazepines in the brain and accumulate secondarily to liver failure. However, additional studies in humans are lacking in order to confirm the link between intestinal flora, liver function, natural benzodiazepines, and HE. An alternative source of natural benzodiazepines could be hemoglobin; metabolites of hemoglobin that mimic benzodiazepines have been described [32].

### 2.5. Manganese

Manganese is probably involved in the development of parkinsonian manifestations in HE, but its role in other neurological manifestations is uncertain [33]. The concentration of manganese is elevated in plasma of patients with cirrhosis and in the brain of patients who die with HE. Hypermanganesemia is the result of portal-systemic shunting and reduction in biliary excretion [34]. Patients with cirrhosis typically exhibit a hyperintense signal in the globus pallidus that has been attributed to the preferential accumulation of manganese in basal ganglia. However, some studies have failed to show a good association between the intensity of the signal in basal ganglia and neurological manifestations of HE [35]. Nevertheless, similarities to the clinical and radiological features of manganese intoxication suggest that the increase in manganese in cirrhosis causes the extrapyramidal signs of chronic HE through mechanisms that impair dopaminergic neurotransmission. The effect of manganese removal on the neurological signs and symptoms of chronic HE has not been evaluated.

### 2.6. Astrocytes

Experimental and pathological evidence point at astrocytes as the main neurological cell affected in [36]. The distinctive morphologic alteration is the Alzheimer type II astrocytic change, which is characterized by a cell with enlarged, pale nuclei with peripheral margination of chromatin and often prominent nucleoli. Results of microscopic studies of specimens from humans and experimental preparations suggest that the astrocytic changes are due to cellular swelling.

Astrocytes occupy one third of the volume of the cerebral cortex. Their foot processes surround brain capillaries, where they contribute to blood brain barrier function. This anatomical organization forms a syncytium, where critical metabolic supportive functions involved in the maintenance and regulation of the extracellular microenvironment, such as uptake of ions and neurotransmitters, influence neuronal excitability and neurotransmission. A specific astrocyte function is the detoxification of ammonia through the amidation of glutamate to glutamine.

Recent studies have emphasized a role for oxidative stress, including nitrosative stress, as a critical factor in ammonia-induced cell impairment through collapse of the inner mitochondrial membrane due to the opening of the permeability transition pore. The collapse leads to mitochondrial dysfunction, energy failure, and additional free radical production. The mitochondrial permeability transition is a Ca2+ dependent, cyclosporine A (CsA) sensitive process due to the opening of a pore in the inner mitochondrial membrane that leads to a collapse of ionic gradients and ultimately to mitochondrial dysfunction. Through this mechanism, ammonia may result in astrocyte dysfunction and HE [24, 37–39].

### 2.7. Neurotransmitter Systems

The improvement of neurological manifestations after the administration of a drug that interacts with an individual transmitter system is an important argument to support a pathogenic role for that system. The first attempt to normalize the abnormalities of neurotransmission arose from the false neurotransmitter hypothesis. The notion was to restore the abnormalities in the profile of plasma amino acids and the transport of amino acids across the blood brain barrier by administering branched chain amino acids. Subsequent therapeutic attempts have been focused on the brain itself and include stimulation of dopaminergic transmission with bromocriptine or levodopa [40] and blockage of GABA inhibitory neurotransmission with flumazenil [30]. The results have not been remarkable, highlighting the complexity of a paradigm where several neurotransmitter systems are simultaneously affected. Still these attempts indicate that it may be possible to treat hepatic encephalopathy using drugs that act in the brain in addition to measures that decrease the plasma level of a putative toxin.

Multiples abnormalities of neurotransmitter systems have been described in HE. Glutamate neurotransmission, which is clearly disturbed in animal models of HE [41], has been proposed to play a role in the pathogenesis of HE. However, supportive human data arise mostly from autopsied samples [42] and pharmacological manipulation of glutamatergic neurotransmission has not been attempted.

Decreasing the neurotransmitter activity will cause a decrease in consumption of oxygen and glucose is accompanied by a parallel decrease in cerebral blood flow [43]. Some studies with humans have shown focal reductions of glucose utilization that have been related to specific neurologic manifestations [44]. However, the findings cannot separate whether the decrease is the cause or the consequence of the encephalopathy. The current interpretation is that, as in other metabolic encephalopathies, the decrease in energy consumption is secondary to the decrease in demand. An increase in supply, as observed in some patients with high cerebral blood flow, especially among those with fulminant hepatic failure, does not improve the mental state [45].

### 2.8. Brain Edema

Brain edema is a complication of fulminant hepatic failure, which can progress to intracranial hypertension and death. Brain edema has been frequently regarded as a distinct entity, dissociated from the neurological features of HE. However, several lines of evidence relate brain edema to HE [46]. Although intracranial hypertension is a common problem in patients with fulminant hepatic failure in coma, the development of high intracranial pressure (ICP) in patients with cirrhosis in deep coma is only occasionally documented [47].

Brain edema has been described in all situations of acute hyperammonemia and has been associated to plasma levels of ammonia in fulminant hepatic failure [23]. In the experimental setting, brain swelling secondary to ammonia infusion can be prevented with the administration of an inhibitor of the synthesis of glutamine. Other factors, such as hyponatremia, may enhance the effects of ammonia on brain swelling [39]. In fulminant hepatic failure, an additional factor that plays an important role in the development of intracranial hypertension is the presence of abnormalities of cerebral circulation. Cerebral vasodilatation and loss of autoregulation are characteristic findings in fulminant hepatic failure [45]. The mechanism that causes the abnormalities of cerebral circulation has not been fully elucidated. They appear to arise from a signal generated in the brain. Indeed, measures that decrease cerebral vasodilatation are of clinical benefit for patients with severe intracranial hypertension [48]. In addition to differences in the cerebral circulation and in the rate of exposure of the brain to ammonia, patients with cirrhosis may activate compensatory mechanisms that counteract osmotic changes in the brain [46]. Those with hyponatremia are at higher risk for the development of intracranial hypertension.

## 3. Clinical Features

### 3.1. Classification

The first step in the approach of HE is to establish the presence or absence of underlying hepatic injury as well as the degree of this injury.

The absence of hepatic cirrhosis leads to rule out the presence of portal-systemic shunts being this vascular disorder the target of the treatment. HE associated to acute liver failure confers a poor prognosis and it is the manifestation of the end stage of this clinical condition involving prognostic and therapeutic decisions. HE associated with cirrhosis and portal hypertension can appear as an isolated episode with or without precipitating event if the episodes resort in time with a period free of event, it is defined as recurrent HE; if it persist over time, it is defined as chronic HE. More recently, another type of HE without apparent clinical disturbance that required psychometric test for diagnose was defined as minimum HE [49] (Table 1).

### 3.2. Acute Episode of Hepatic Encephalopathy

Acute HE can appear as an isolated decompensation without worsening of the hepatic function, as part as an acute-on-chronic liver failure or as part as an acute liver failure without previous hepatic disease. A special feature is the appearance of acute HE in the context of portal-systemic shunts with or without liver disease. Each specific case confers a different prognosis; when the HE episode appears in the context of acute-on-chronic liver failure a poor prognosis has been observed (cumulative mortality of 55% in 9 month) in contrast to when it occurs as a simple decompensation, the mortality was lower 25%. Acute liver failure in the absence of cirrhosis, deserve a special mention, this entity has a high mortality and usually requires a liver transplant.

The acute HE episode could be associated with a precipitating factor but in the majority of cases no precipitating factor was identified.

An acute episode of HE is characterized by the development of an acute confusional syndrome that includes impaired mental state, neuromuscular abnormalities, fetor hepaticus, and hyperventilation [2]. Variability is an important feature; the clinical manifestations may fluctuate very rapidly and oscillate from mild confusion to deep coma. The onset is usually abrupt; HE develops over hours to days. Most patients do not have significant neurological manifestations before the onset of the acute episode of HE, unless they had persistent HE. The evolution of an acute episode of HE tends to parallel the course of liver function or the removal of the precipitating factor. Patients usually recover from HE without major neurological deficits and are able to return to previous activities.

Impairment of consciousness initially manifests as subtle changes of personality or disturbances in the circadian rhythm of sleep and wakefulness (insomnia during the night and somnolence during the day). As HE progresses, the manifestations include inappropriate behavior, disorientation, confusion, slurred speech, stupor, and coma. Some patients may experience nausea and vomiting, especially if there is a rapid evolution into coma.

Asterixis is a characteristic feature of HE that represents the failure to actively maintain posture or position [2]. Asterixis is caused by abnormal function of diencephalic motor centers that regulate the tone of agonist and antagonist muscles, normally involved in maintaining posture [50]. The classic method of eliciting asterixis is by dorsiflexion of the patient's hand with the arms outstretched and fingers separated. The postural lapse that occurs consists of a series of rapid, involuntary, flexion-extension movements of the wrist. Asterixis may be observed during any sustained posture: tongue protrusion, dorsiflexion of the foot, or fist clenching. Asterixis is not exclusive to HE and can occur in other metabolic or structural encephalopathies (renal failure, hypercapnia, and stroke affecting basal ganglia). Asterixis does not occur in early or advanced HE. In coma, asterixis disappears, but the patient may exhibit signs of pyramidal involvement, such as exaggerated deep tendon reflexes, hypertonia, or extensor plantar responses. Transient decerebrate posturing and abnormal ocular movements may occur in deep coma.

Fetor hepaticus is a peculiar pungent odor of the breath that is often regarded as a component of HE. This odor has been attributed to dimethylsulphide, a volatile sulfur compound that can be identified in the breath and serum of patients with cirrhosis [51]. The presence of fetor hepaticus is not constant; patients with cirrhosis without HE can have this condition. Hyperventilation is also frequent, especially among patients with advanced HE. Hyperventilation has been interpreted as a compensatory mechanism that decreases the entrance of ammonia into the brain via a decrease in arterial pH. It has also been related to elevated levels of estrogens and progestogens [52].

### 3.3. Chronic Encephalopathy

Chronic encephalopathy encompasses two different situations: (a) the patient with relapsing episodes of HE and (b) the patient with persistent neurological manifestations. This differentiation highlights the more prominent clinical presentation, but in practice both situations are difficult to separate. Some patients initially have relapsing episodes and later have persistent symptoms. A patient with pure relapsing HE or pure persistent HE is rare. Furthermore, symptoms tend to fluctuate after the institution of therapeutic measures or the occurrence of precipitating events.

Relapsing episodes may be due to precipitating factors, but in the majority of cases they are spontaneous or related to discontinuation of medication. A history of constipation is commonly elicited. The course of the acute episode does not differ from the one previously described, except for a tendency for an abrupt onset and resolution. Between episodes, the patient can be perfectly alert and not showing any sign of cognitive dysfunction. However, a careful neurological exam and neuropsychological tests may reveal abnormalities. Mild parkinsonian signs, characterized mostly by bradykinesia without tremor [53], are probably the most common manifestation between episodes.

Persistent HE refers to those manifestations that do not reverse despite adequate treatment. In the majority of patients with cirrhosis and prior episodes of acute HE and advanced liver failure, a careful neurological examination may reveal multiple mental and motor abnormalities. Most of these abnormalities are subtle, such as increased muscle tone, reduced mental or motor speed, dysarthria, hypomimia, lack of attention, or apraxia. Psychometric tests may be helpful in describing and quantifying the degree of impaired mental function.

Persistent HE is considered severe when it impairs daily activities. The most characteristic manifestations of severe chronic HE are dementia, severe Parkinsonism, or myelopathy in combination with other manifestations of neurological involvement (ataxia, dysarthria, gait abnormalities, and tremor). This clinical picture is seldom seen nowadays, as a result of the availability of liver transplantation and the limited number of patients that undergo surgical portal-systemic shunts. Patients with* hepatic dementia* tend to have fluctuating symptoms with periods of improvement and a subcortical pattern. The initial manifestations are attention deficits, visuopractic abnormalities, dysarthria, and apraxia. Those with* hepatic Parkinsonism* may resemble Parkinson's disease, except for a symmetrical presentation and lack of significant tremor.* Hepatic myelopathy* [54] is characterized by a progressive spastic paraparesis accompanied by hyperreflexia and extensor plantar responses. Only a few patients have sensory symptoms or incontinence. The pathogenetic mechanisms of these complications are obscure. They have associated neuronal loss, in the case of dementia, and demyelination along the pyramidal tract in the case of myelopathy. Although these lesions are difficult to reverse, there have been descriptions of improvements after liver transplantation [55], a challenge to the notion of irreversibility. The term* hepatocerebral degeneration* has been occasionally used to describe such patients. However, this is a neuropathological diagnosis applied to those brains that exhibit substantial and irreversible loss of gray matter in cortex and basal ganglia. It is preferable not to use it to describe the clinical picture.

### 3.4. Fulminant Hepatic Failure

The clinical picture of HE in acute liver failure parallel that of an acute episode of HE, an acute confusional syndrome that evolves into coma. However, in acute liver failure, brain edema leading to intracranial hypertension and abnormalities of brain perfusion is critical [56].

Brain edema does not result in clinical manifestations unless intracranial hypertension is present, as the displacement of brain tissue is the factor that results in neurological symptoms. Intracranial hypertension may manifest as decerebrate rigidity, myoclonus, seizures, mydriasis, bradycardia, or arterial hypertension (the Cushing reflex). However, the diagnosis of intracranial hypertension based on clinical signs in unreliable, as they can be absent with pressures as high as 60 mm Hg [57] and difficult to monitor because these patients are intubated and paralyzed when they are in coma.

A major consequence of intracranial hypertension is the effect on cerebral perfusion. The maintenance of cerebral blood flow is critical to assure an adequate supply of oxygen. The driving force in maintaining a stable blood flow is the cerebral perfusion pressure, the arithmetical difference between mean arterial pressure and ICP. When cerebral perfusion pressure is less than 40 mm Hg, structural tissue damage from brain ischemia may ensue. In spite of low cerebral blood flow, an occasional patient may recover from this situation without irreversible brain damage. Another consequence of intracranial hypertension is the mechanical compression of neighboring structures. The increase in pressure causes displacement of brain tissue, resulting in herniation and direct compression of the temporal lobe or the cerebellum. Brain stem compression can result in sudden respiratory arrest and circulatory collapse.

### 3.5. Minimal Hepatic Encephalopathy

*Minimal* HE, also referred to by the terms* latent* or* subclinical*, is a mild dysfunction of brain function that cannot be detected by standard clinical examination [2, 3, 58]. This label was originally applied to a group of individuals who performed abnormally on psychometric tests but had essentially normal findings at clinical examination. Psychometric tests are more sensitive than clinical observation, as shown in other neuropsychiatric diseases, such as dementia.

Other techniques (EEG, evoked potentials, and neuroimaging) that are more sensitive than clinical examination to reveal neurological impairment have also shown a stage of minimal dysfunction. This stage is understood as part of a continuous disorder that has several levels of severity, with minimal HE being the mildest expression of HE. This interpretation is supported by the observation of improvements of minimal HE after the same therapeutic measures that are addressed against overt HE [59] and the relationship between minimal HE, ammonia levels, and liver function [60].

The diagnosis of minimal HE is arbitrary and can be performed with neuropsychological or neurophysiological tests. The most characteristic deficits are in motor and attention skills [61]. Learning impairment, which has also been described in experimental models [62], appears to be the consequence of attention deficits [63]. The depth of the psychometric and the clinical examination necessary to diagnose minimal HE is not defined. The frequency of the diagnosis is very variable (30% to 84% of patients), depending on the characteristic of the population being studied and the extent of the psychometric evaluation. Some attempts have been made to develop practical tools based on the design of short batteries of neuropsychological tests, such as the* PHES* [64]. However, these batteries have not been fully standardized and their use is still investigational. Critical flicker frequency, a neurophysiological tool, has been proposed as a practical test to assess low grade encephalopathy [65].

The importance of establishing the diagnosis of minimal HE is unknown. Some studies have highlighted that minimal HE may have an adverse impact on the ability to perform daily activities and on health-related quality of life [66, 67]. However, many subjects are able to compensate for these deficits [58]. From a practical point of view, a psychometric evaluation may be adequate in those individuals whose occupations demand attention and motor abilities. A report of impaired driving in patients with minimal HE [68] suggests the need to develop a therapeutic program for such individuals. Benefits of the treatment, assessed by monitoring the neuropsychological response, should be weighed against secondary side effects. There are no data on the effects of therapy on health-related quality of life. Patients with cirrhosis and minimal HE have a clear tendency to develop overt HE [69]. Whether the institution of preventive measures may decrease the risk of the progression to overt encephalopathy has not been evaluated. The presence of minimal HE indicates worse prognosis, especially if associated with high blood ammonia after the administration of glutamine [70].

## 4. Methods for the Assessment of Hepatic Encephalopathy

### 4.1. Grading Hepatic Encephalopathy

Grading of HE is necessary to assess the evolution of the condition and the response to the effects of therapy. Several methods are based on clinical findings or the combination of neurophysiological and neuropsychological tests, but the simplest grading of HE is based on clinical findings. The West Haven index is widely used [71]. It is based on changes in consciousness, intellectual function, and behavior (Table 2). The Glasgow coma scale offers a system to monitor consciousness according to simple and more objective parameters. This scale was initially developed for traumatic coma but has gained widespread use for all forms of coma. It is probably more reliable than the West Haven criteria but has the limitation that it is less sensitive in quantifying the mildest forms of HE and is better suited for advanced HE.

There are other scales widely used like the Clinical Hepatic Encephalopathy Staging Scale (CHESS); this scale evaluated nine different items with a dichotomic answer, giving a punctuation from 0 (no HE) to 9 (deep coma); the principal disadvantage is the fact that the CHESS does not evaluate the asterixis (Table 3) [72].

The portal-systemic encephalopathy index (PSE index) has been used in many studies to assess the effects of therapeutic measures. This index combines the assessment for mental state, arterial ammonia, electroencephalography, the number connection test, and estimation of the degree of asterixis. An arbitrary weight of 3 is assigned to the mental state and the other parameters are weighted. Concerns have been raised about the arbitrary scoring system, the inclusion of ammonia (a putative toxin), the feasibility of an arterial puncture, and the assessment of the number connection test in the evaluation of advanced HE. It is generally considered that blood levels of ammonia, though separating groups according to mean values [73], show wide dispersion in individual values and are not useful to predict the severity of HE and to monitor the response to therapy [74]. A consensus has been reached indicating that the PSE index is not adequate for clinical follow-up and is not recommended for clinical trials.

### 4.2. Neuropsychological Tests

The main role of neuropsychological tests is the diagnosis of minimal HE and the assessment of cognitive function in patients with persistent HE. Based on the most frequently found abnormalities, several psychometric tests have been proposed to be the most adequate for diagnosing HE [58, 60]. Neuropsychological tests can be affected by multiple factors. It is important that the neuropsychological assessment takes into consideration these factors. Patients with clear signs of decreased arousal cannot undergo testing. Care is needed to control comorbidities, visual impairment, or cultural barriers. The test should be adapted to the cultural characteristics of the population being evaluated. Nomograms to compare the results should take into consideration age and ideally the degree of education. The patient undergoing testing should be sitting in a quiet room with sufficient light. An important limitation of the neuropsychological tests is the practice effect in follow-up evaluation. Results of psychometric tests are affected by learning. Use of parallel versions can lessen this effect, but only few tests have well-standardized versions.

Some short batteries specifically developed for HE may be useful for the detection of abnormal cognition. However, they do not substitute a formal neuropsychological evaluation performed by an experienced neuropsychologist. ThePHES is a battery of tests, specifically developed for the diagnosis of minimal HE [75]. Similar to the mini-mental state examination for dementia, it can be useful for screening. The PHES combines 5 paper-pencil tests (line tracing tests, digit symbol test, serial dotting test, number connection test A, and number connection test B) that examine motor speed and accuracy, visual perception, visual-spatial orientation, visual construction, concentration, attention, and to a lesser extent memory. The results of the battery are scored according to nomograms from a group of healthy controls. A pathological test result (diagnosis of minimal HE) has been set at −4 points, http://www.redeh.org/phesapp/datos.html.

Another useful test is the critical flicker frequency; this test has the advantage that it is not conditioned by the cultural or education level but it is necessary that the patient have nonvisual impairment. It consists of placing a flashing light in the visual field of the patient using specially designed glasses. The light flashes initially at a high frequency, so it seems a constant light. Gradually, the frequency decreases, and the light becomes intermittent; that moment has to be identified by the patient pressing a button [75, 76].

### 4.3. Neurophysiologic Tests

A large number of different neurophysiological tests have been proposed for the diagnosis and quantification of HE. Reports for and against the specificity of electrophysiological changes have been published [77, 78]. These tests are most useful in documenting cerebral dysfunction in difficult cases and possibly in monitoring response to therapy. They have the advantage of not being influenced by learning effects. Thus, they may be better suited for assessing effects of treatment than neuropsychological tests, especially for advanced stages of HE. For minimal to mild HE, neurophysiological tests do not give information about behavioral consequences, in contrast to the insight provided by neuropsychological tests.

The standard electroencephalogram (EEG) shows slowing of the frequency from the normal 8–13 Hz to the delta range below 4 Hz. The change usually commences in the frontal or central regions and progresses posteriorly. High voltage, low-frequency (1.5 to 3 Hz) waves with triphasic appearance have been considered characteristic for HE. However, they have been described in a variety of forms of metabolic encephalopathy and are not specific to HE. Several stages of evolution of EEG changes have been described in HE and a fair correlation with clinical stages and ammonia levels has been observed. The simplest EEG assessment is to grade the degree of abnormality of the conventional tracing. Computer-assisted frequency analysis of the EEG includes evaluation of the mean dominant frequency and the power of a particular EEG rhythm. Minor changes in the dominant frequency occurs in patients with minimal HE [59].

Evoked responses are externally recorded potentials reflecting discharges through neuronal networks after exposure to specific stimuli [79]. Depending on the type of stimulus and the pathway analyzed, they could be visual, somatosensory, or acoustic evoked potentials. Event-related potentials using different stimuli represent endogenously task-related cortical response reflecting the neural pathway involved in awareness, learning, and decision-making processes. Event-related potentials, such as the P300 evoked potential, requires patient cooperation and well-trained operators. Evoked potentials and event-related potentials are considered more sensitive than the conventional EEG for the diagnosis of mild forms of HE. They may be useful for assessing the presence of minimal or mild HE in patients with cirrhosis who have memory loss or other mental symptoms.

### 4.4. Neuroimaging

At autopsy, the brains of cirrhotic patients dying in HE do not show major anatomic abnormalities, except for various degrees of atrophy. Thus, neuroimaging studies that assess exclusively the morphologic structure of the brain, such as computed tomography (CT), do not detect specific abnormalities in HE. Brain atrophy, which is depicted with CT, is more common in patients with longstanding cirrhosis and chronic HE [80]. However, brain atrophy is not a specific abnormality of HE and may be related to other factors than HE (alcoholism, age, and comorbid conditions). Furthermore, as in other neurodegenerative diseases, brain atrophy is not associated with neuropsychological performance [79]. Conventional neuroimaging techniques are insensitive in the detection of brain swelling that may develop in some patients with cirrhosis and frequently complicates HE in acute liver failure [81]. No studies have been able to find a neuroimaging correlation of hepatocerebral degeneration (cortical laminar necrosis and polymicrocavitation at the corticomedullary junctions and in the striatum).

Magnetic resonance imaging (MRI) and spectroscopy allow the acquisition of data on cerebral metabolic function that are otherwise not available [29]. Proton MRI shows a typical pallidal hyperintensity on T1-weighted images. This abnormality is most frequently seen in patients with cirrhosis with severe liver failure or long-standing portal-systemic shunts and is absent or only minimally present in patients with well-compensated cirrhosis and unimpaired neuropsychiatric function. It can be also present in patients with congenital shunts or portal thrombosis and normal liver function [51]. No direct correlation between the magnitude of pallidal hyperintensity and the grade of HE have been found, but some studies have related pallidal hyperintensity to parkinsonian manifestations [82]. Because of radiologic similarities to manganese intoxication, it has been proposed that pallidal hyperintensity is the consequence of the preferential deposition of manganese in the basal ganglia. The deposition of manganese in brain tissue would be secondary to portal-systemic shunting and might be involved in the parkinsonian symptoms found in persistent HE.

Proton magnetic resonance spectroscopy allows the assessment of several brain metabolites (glutamine, glutamate, myoinositol) that may be related to the pathogenesis of HE. The level of glutamine, the product of ammonia metabolism in astrocytes, is characteristically increased in brain tissue. Although glutamine is considered neuronally inactive, changes in its concentration may affect neuronal-astrocytic trafficking and interact with glutamate neurotransmission [36]. The concentration of glutamine in cerebrospinal fluid, an indicator of its level in brain tissue, has been correlated to the stage of HE. Unfortunately, the standard available systems, in which magnetic fields of 1.5 Teslas are used, do not allow a separation between the peaks of glutamine (moderate to high increase in HE) and glutamate (mild decrease in HE). Myoinositol has an important role in osmotic regulation in astrocytes. The decrease in brain myoinositol found by spectroscopy has been corroborated in experimental preparations and has been attributed to a compensatory response to the increase in intracellular osmolality caused by the increased concentration of glutamine [46]. Although the technique is insensitive to mild changes in the concentration of metabolites, the abnormalities found with spectroscopy have been related to neuropsychological impairment and to liver function [83]. The role of proton magnetic resonance in the diagnosis of HE has not been investigated. Nevertheless, a completely normal study in a patient suspected to suffer from HE is a strong argument against this diagnosis.

Regional distribution of radionuclides in the brain has been used to study cerebral blood flow, oxygen and glucose consumption, neurotransmitter utilization, and availability of neuronal receptors. The results of some of these studies are controversial [84]. Although they may help in the understanding of the pathogenesis of HE, radionuclide studies are not adequate for diagnostic purposes.

## 5. Principles of Treatment

### 5.1. General Treatment

Due to the lack of a pathogenetic mechanism that explains the whole syndrome, therapy is focused on correcting the precipitating factors and decreasing plasma levels of ammonia and related metabolites. Most data have not been evaluated in randomized clinical trials with large number of patients. Study design is especially complex in this condition, as the clinical course of HE tends to resolve and relapse spontaneously in many cases. The concurrence of other disorders (anemia, electrolytic disturbances, fever, severe infection, and alcoholic injury) is a confounding factor that complicates the assessment of the neurological manifestations. For these reasons, many modalities of therapy have been criticized. Nevertheless, in recent years there have been several studies that render important data and that have changed the management of the patient that develops HE.

The approach to the patient with overt HE must guarantee the vital support of the patient, correct the precipitating event, and withdraw diuretics. The treatment of HE has different targets that are responsible for the plasma rise of toxic substances with potential effects on central nervous system, with ammonia being the most relevant [85]. The best results have been obtained in studies focused on prevention of recurrence with lactulose, rifaximin, and HPN-100. Use of these drugs in the episode of overt HE is extrapolated from preventive studies.

### 5.2. Nutrition

Classically, the recommendation for patients with HE has been to restrict dietary protein intake, but this is a measure that nowadays is considered wrong. For several decades it was recommended withholding all protein intake and subsequently increasing the intake to maximal tolerance [86]; however, this recommendation has no solid support [87]. Only one randomized study has investigated the effects of protein restriction on the outcome of HE [88]. In this study, thirty patients admitted for an episode of HE received progressive amounts of proteins (from 0 to 1.2 g·kg·d) or normal protein amounts (1.2 g·kg·d) from the beginning. The diet was administered via nasogastric tube for 2 weeks and HE was assessed blinded for group of treatment. The main result of the study was that there were no differences in the outcome of HE, while the normal protein diet resulted metabolically to be more adequate. Thus, restriction of proteins of the diet does not appear to have any beneficial effect for episodic HE. From this study the nutritional management of patients with HE has changed radically. Although avoiding intake of large amounts of protein may be positive to reduce toxins that are involved in HE, restriction may worsen liver function and increase the risk of death. A positive nitrogenous balance may improve encephalopathy by promoting hepatic regeneration and increasing the capacity of muscle to detoxify ammonia. For these reasons the current recommendation is to avoid restrictions of dietary protein [87]. Patients are nowadays treated with nutritional support that provides at least a dose of protein of 0.8 gr/kg/d.

Some studies suggest that the type of protein may influence the outcome. Branched chain amino acids were promoted as a mechanism to correct the imbalance in the plasma amino acid profile and thought to be involved in the pathogenesis of hepatic encephalopathy. However, clinical trials using branched chain amino acids have not shown dramatically beneficial effects for episodes of HE. A new analysis of several published studies, including some more recent, supports a mild positive effect of BCAA on minimal HE, whose clinical impact is debatable [89, 90].

Branched chain amino acids do not show significant effects on survival. Critical reviews of the published studies highlight the inadequate design of the majority of studies. Considerations of cost-effectiveness indicate that branched chain amino acids should not be used outside clinical trials [91]. Branched-chain amino acids show anticatabolic effects in patients with chronic liver diseases, probably due to their ability to serve as an energy substrate for muscle and because of their actions on muscle protein synthesis and degradation. This nutritional effect may result in an improvement of liver function and a better clinical outcome, as shown in a multicenter trial performed in Italy that included patients with advanced cirrhosis, most of them without prior HE [92], most recent articles has shown no improvement in recurrence of overt HE but has been effective in increasing muscle mass and improving MHE [89].

### 5.3. Nonabsorbable Disaccharides

Lactulose (*β*-galactosido-fructose) and other nonabsorbable disaccharides were introduced with the goal of promoting the growth of* Lactobacillus bifidus*, which contains little urease activity and via this mechanism could decrease the production of ammonia in the colon. This is not its mechanism of action, which is still complex and not fully understood. The bulk of evidence links the efficacy of lactulose to an interaction with the enteric flora and to a decrease in the generation of nitrogenous compounds in the intestine [93]. Administered orally, lactulose is not broken down by intestinal disaccharidases and reaches the cecum, where it is metabolized by enteric bacteria to lactate and acetate [94], causing a drop in cecal pH leading to an increase in fecal nitrogen excretion and a decrease in the amount of nitrogen that reaches portal blood [95].

The efficacy of lactulose has been demonstrated in the primary and secondary prevention of hepatic encephalopathy. Two studies conducted in India showed marked improvement in the recurrence of HE after approximately one year of treatment with lactulose. The results of these studies have led to considering the use of lactulose as a standard of care. The efficacy is shown in the prevention, but its use has been generalized to its treatment. Singularly, it is not known to what extent the effect of lactulose is simply as a laxative and whether other laxatives have similar benefits. There have been additional studies showing a positive effect of nonabsorbable disaccharides in HE, which were reviewed by Als Nielsen et al. in 2004 [96]. While the studies showed an improvement of HE, their quality was considered insufficient. Thus, while there are no doubts in using lactulose or lactitol for preventing HE, it would be recommendable to perform new studies with nonabsorbable disaccharides to show their efficacy in the treatment of episodic HE. The use of disaccharides is further supported on biological foundations and extensive experience of use [2]. Treatment of minimal HE has shown in several studies an improvement of neuropsychological tests and quality of life indexes. However, the impact on clinically relevant endpoints is a matter of debate and treatment of minimal HE is not firmly established [96].

### 5.4. Antibiotics

The use of antibiotics in the treatment of HE is based on its action in the intestinal flora. Apart from decreasing the bulk of intestinal flora, different mechanisms have been proposed; they include inhibition of the activity of intestinal glutaminase (enzyme that is activated in cirrhotic patients) and through this mechanism decrease plasma ammonia [97]. Another action is decreasing bacterial translocation and downregulating the immune response triggered by these bacterial products [98].

Neomycin was the first antibiotic that was tested in the treatment of HE that showed similar efficacy to lactulose in chronic HE [99]. Since neomycin is an aminoglycoside, the major concern with its use is the potential of renal or auditory toxicity. Absorption of neomycin is poor (<4%) and the drug is considered potentially toxic only after long-term use. Other antibiotics that were proposed were metronidazole and vancomycin; their potential toxicity have led to abandon them, especially in long-term use.

Rifaximin is an antibiotic with wide antibacterial action and low intestinal absorption that is currently recommended for the prevention of HE, especially because it has minimal toxic effects that allow prolonged use. Studies that have compared rifaximin (1200 mg/d) to nonabsorbable disaccharides (lactulose 45 to 120 mL/day, lactitol 60 mg/day) [100–106] show a slightly better, but not significant, outcome for rifaximin. Similar effects were also seen when comparing rifaximin to neomycin (4500 mg o 3000 mg) [107–110] or paromomycin (1500 mg/d) [111]. Significantly, only three of these studies [100, 104, 106] were performed during an overt episode of HE and none of them had a placebo group. Eltawil et al. [112] included all these studies in a meta-analysis; the comparison of rifaximin and disaccharides found that both groups reach the primary endpoint (recovery of the HE episode or clinically relevant improvement) without differences (OR = 1.92, 95% IC: 0.79–4.98, and *P* = 0.15). Similar results were found when comparing rifaximin with other antibiotics (OR = 2.77, 95% IC: 0.35–21.83, and *P* = 0.21). No differences were also found comparing rifaximin to nonabsorbable disaccharides or to other antibiotics (paromomycin or neomycin). Nevertheless, there was a trend towards a better outcome in favor of rifaximin (OR = 1.96, 95% IC: 0.94–4.08, and *P* = 0.07) [112]. Again no statistical differences were observed in plasma ammonia [100, 104, 106, 108–111] or improvement of psychometric tests [102, 106, 110] but rifaximin exhibited a better safety profile (OR = 0.27, 95% IC: 0.15–0.59, and *P* < 0.01) based on a lower frequency of diarrhea.

One study has compared rifaximin to placebo in the secondary prevention of recurrent HE (at least 2 episodes in 6 months). Since these patients corresponded to a highly recurrent population, almost all patients (90%) were receiving in addition lactulose. The more important result is that those that received rifaximin had a much lower number of new episodes of HE in the following 6 months (22% versus 46%). Hospitalizations associated with HE was also lower in the rifaximin group (13% versus 22%) [113]. Furthermore, an analysis of the quality of life of these patients showed that those with recurrent HE had lower scores on daily life activities [114]. Based on these results, it is common to recommend the use of lactulose or lactitol after the first episode of HE, in combination with rifaximin after the second episode. It is not known whether rifaximin will also be necessary in the prevention of recurrence in patients with well-defined precipitating events that have been resolved (i.e., severe infection, dehydration, and renal failure).

In summary, rifaximin has been shown to be effective in the prophylaxis of new HE episodes with dose 1200 mg/d combined with lactulose, but no studies comparing its efficacy to placebo have been performed for assessing its effectiveness during the episode of HE and no difference with nonabsorbable disaccharides or other antibiotics has been demonstrated in this clinical situation.

### 5.5. Modulators of Ammonia Metabolism

Glycerol phenylbutyrate (HPN-100) is a prodrug of phenylbutyric acid (PBA), which is absorbed from the intestine and converted by way of b-oxidation to the active moiety, phenylacetic acid (PAA). PAA is conjugated with glutamine in the liver and kidney by way of N-acyl coenzyme A-L-glutamine N-acyltransferase to form phenylacetylglutamine (PAGN) and hydrosoluble molecule that could be eliminated trough the urine. This molecule has proven its safety in cirrhotic patients [115]. In a recent randomized double-blind study, the administration of a HPN-100, which enhanced the urinary excretion of ammonia equivalents, resulted in a lower number of recurring HE episodes. A significant number received rifaximin in addition to HPN-100. In this subgroup, the combination of rifaximin and HPN-100 did not show better results than isolated rifaximin [116].

Ornithine phenylacetate (OP) is a new proposal for decreasing ammonia in cirrhosis trough the combined administration of PA and ornithine. This therapy is based on the capacity of ornithine to stimulate the activity of glutamine synthetase; hence, incorporating ammonia into a “nontoxic” molecule. The newly formed glutamine will combine with PA allowing the elimination of ammonia in the urine by its conversion into PAGN. This strategy prevents the degradation of glutamine by intestinal glutaminase and avoids new formation of ammonia [117]. This molecule has demonstrated to be safe in decompensate cirrhotic patients and the current data seems to confirm its efficacy in decreasing plasma ammonia [118].

### 5.6. Detoxifying Therapies

Extracorporeal albumin dialysis has been proposed for improving the survival and treating cirrhotic patients with severe decompensation or acute or chronic liver failure (ACLF) [119]. There have been two trials with the goal of reducing mortality of severely decompensated cirrhosis: The RELIEF trial [120] and the HELIOS trial [121]. Both were randomized studies that included a large number of patients (*n* = 145 and *n* = 189). Therapy with extracorporeal systems did not show significant differences in survival at 4 weeks. However, there were some positive indicators (bilirubin, renal function, and degree of hepatic encephalopathy) that support that these therapies may have a niche in specific group of patients. A study using the molecular adsorbent recirculating system (MARS) found a more rapid improvement in the severity of HE, which has been the basis for its approval for patients with severe HE [120]. Albumin has also been proposed for onset HE in addition to laxatives and rifaximin; however, recently, there were no differences in the outcome of HE between albumin and placebo; however, survival in the follow-up was better in the albumin group at 90 days [122].

### 5.7. Special Cases

Patients with a good liver function (MELD < 12) and large portal-systemic shunts should be evaluated for possible occlusion of the shunt using angioradiological embolization, which has been shown safe and effective [123]. Embolization is tolerated in cirrhotic associated shunts but should not be performed in congenital hypoplasia of the portal vein to avoid massive upper gastrointestinal bleeding post embolization [124].

Special attention must be given to patients who have undergone placement of a transjugular intrahepatic portosystemic shunt (TIPS); one of the most relevant adverse events is overt HE, which usually appear in 33% of the patients during the first month after the placement. Some factors have been associated with a higher HE risk (age over 65 years, previous episodes of overt HE, and Child-Pugh C) but prophylactic use of nonabsorbable disaccharides or rifaximin have not shown to be effective during the first month after TIPS. For this reason antiencephalopathy therapy is usually reserved for the symptomatic patient [125].

## Figures and Tables

**Table 1:** Classification of hepatic encephalopathy.

HE associated to an acute liver failure					

HE associated to a portosystemic shunt without hepatic cirrhosis					

HE associated to cirrhosis and portal hypertension					

Episodic		Chronic		Minimum	
*↙*	*↘*	*↙*	*↘*		
Primary	Secondary	Mild	Severe		

HE: hepatic encephalopathy.

**Table 2:** The West Haven scale.

Stage	The West Haven criteria	Adapted West Haven criteria
0	No abnormality detected	**Alert and attentive** (oriented in time and space) without signs of encephalopathy (neither dysarthria, ataxia, flapping tremor, or obvious decrease in the speed of mental processing)

1	Trivial lack of awareness Euphoria or anxiety Shortened attention span Impairment performance of addition	**Alert and attentive, but with at least one of the following signs**: dysarthria, ataxia, flapping tremor, or obvious decrease in the speed of mental processing

2	Lethargy or apathy Minimal disorientation for time or place Subtle personality change Inappropriate behavior Impaired performance of subtraction	**Awake but inattentive**: disoriented, somnolent, easy to distract, and unable to perform easy mental tests (addition, subtraction, and remember a list of numbers) Patient's speech is easy to understand

3	Somnolence to semistupor but responsive to verbal stimuli Confusion Gross disorientation	**Marked somnolence** or psychomotor agitation Speech is difficult to understand

4	Coma (unresponsive to verbal or noxious stimuli)	**Coma** The patient does not speak and does not follow simple commands (such as raising an arm or opening the mouth)

**Table 3:** CHESS scale.

Medscape http://www.medscape.com/		

Item	Score	
	0	1

(1) Does the patient know which month he/she is in (i.e., January, February)?	Yes	No, he/she does not talk
(2) Does the patient know which day of the week he/she is in (i.e., Thursday, Friday, Sunday, etc.)?	Yes	No, he/she does not talk
(3) Can he/she count backward from 10 to 1 without making mistakes or stopping?	Yes	No, he/she does not talk
(4) If asked to do so, does he/she raise his/her arms?	Yes	No
(5) Does he/she understand what you are saying to him/her? (Based on the answers to questions 1 to 4)	Yes	No, he/she does not talk
(6) Is the patient awake and alert?	Yes	No, he/she is sleepy or fast asleep
(7) Is the patient fast asleep, and is it difficult to wake him/her up?	No	Yes
(8) Can he/she talk?	Yes	He/she does not talk
(9) Can he/she talk correctly? In other words, can you understand everything he/she says, and he/she does not stammer?	Yes	No, he/she does not talk or does not talk correctly
Total score of the CHESS:		

The total score is the sum of the answers to the nine items. Minimal score = 0; maximal score = 9.

Source: Semin Liver Dis © 2008 Thieme Medical Publishers.
